# Venom composition of *Trimeresurus albolabris, T. insularis, T. puniceus* and *T. purpureomaculatus* from Indonesia

**DOI:** 10.1590/1678-9199-JVATITD-2021-0103

**Published:** 2022-07-06

**Authors:** Syahfitri Anita, Arif Rahman Sadjuri, Latri Rahmah, Herjuno Ari Nugroho, Mulyadi, Wahyu Trilaksono, Wiwit Ridhani, Nabila Safira, Hariman Bahtiar, Maharani, Amir Hamidy, Adriansjah Azhari

**Affiliations:** 1 Laboratory of Herpetology, Museum Zoologicum Bogoriense, Research Center for Biosystematics and Evolution, National Research and Innovation Agency (BRIN), Cibinong, Indonesia; 2 Department of Zoology, Graduate School of Science, Kyoto University, Sakyo, Kyoto, Japan.; 3 PT Bio Farma (Persero), Bandung, Indonesia.; 4 Research Center for Applied Zoology, National Research and Innovation Agency (BRIN) , Cibinong, Indonesia.; 5 PT Dermama Bioteknologi Laboratorium, Betshaida Hospital, Tangerang, Indonesia.

**Keywords:** Trimeresurus albolabris, Trimeresurus insularis, Trimeresurus puniceus, Trimeresurus purpureomaculatus, Snake venom, Venom proteome

## Abstract

**Background:**

Several studies have been published on the characterization of *Trimeresurus* venoms. However, there is still limited information concerning the venom composition of *Trimeresurus* species distributed throughout Indonesia, which contributes to significant snakebite envenomation cases. The present study describes a comparative on the composition of *T. albolabris, T. insularis, T. puniceus,* and *T. purpureomaculatus* venoms originated from Indonesia.

**Methods:**

Protein content in the venom of four *Trimeresurus* species was determined using Bradford assay, and the venom proteome was elucidated using one-dimension SDS PAGE nano-ESI- LCMS/MS shotgun proteomics.

**Results:**

The venom of *T. albolabris* contained the highest protein content of 11.1 mg/mL, followed by *T. puniceus*, *T. insularis* and *T. purpureomaculatus* venom with 10.7 mg/mL, 8.9 mg/mL and 5.54 mg/mL protein, respectively. In total, our venomic analysis identified 65 proteins belonging to 16 protein families in *T. purpureomaculatus*; 64 proteins belonging to 18 protein families in *T. albolabris*; 58 different proteins belonging to 14 protein families in *T. puniceus*; and 48 different proteins belonging to 14 protein familiesin *T. insularis.* Four major proteins identified in all venoms belonged to snake venom metalloproteinase, C-type lectin, snake venom serine protease, and phospholipase A2. There were 11 common proteins in all venoms, and *T. puniceus* venom has the highest number of unique proteins compared to the other three venoms. Cluster analysis of the proteins and venoms showed that *T. puniceus* venom has the most distinct venom composition.

**Conclusions:**

Overall, the results highlighted venom compositional variation of four *Trimeresurus* spp. from Indonesia. The venoms appear to be highly similar, comprising at least four protein families that correlate with venom’s toxin properties and function. This study adds more information on venom variability among *Trimeresurus* species within the close geographic origin and may contribute to the development of optimum heterologous antivenom.

## Background

**Figure 1.**
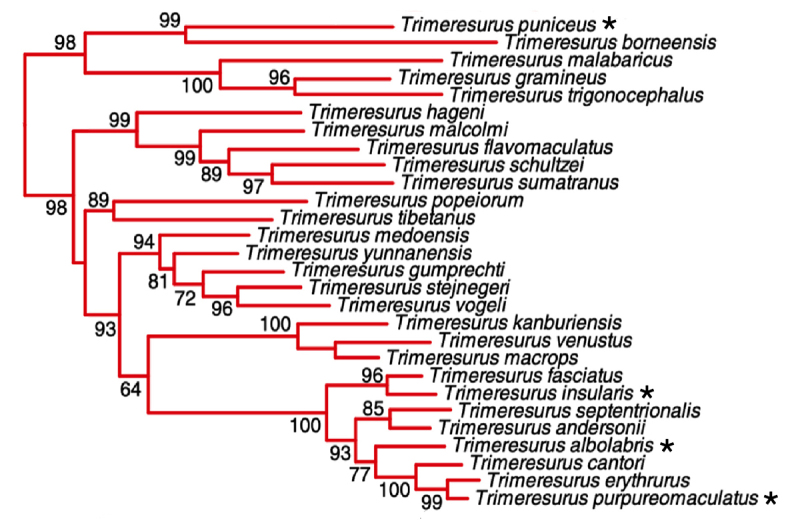
Phylogeny of the Asian pit viper (*Trimeresurus*) complex, adapted from Pyron et al. [6]. Black asterisks mark the species sampled in the present study.

The pit vipers of the genus*Trimeresurus*represent over 40 species of venomous snakes that are morphologically, ecologically and geographically diverse [1-3]. There are 19 species of Crotalinae (pit vipers) distributed in Indonesia, and 13 of them belong to *Trimeresurus* genus [4]. These small to medium-sized snakes have a triangular head that is usually very distinct from the neck and equipped with a pair of long, hinged fangs in the anterior upper jaw. *Trimeresurus* is a nocturnal, bush- and tree-dwelling animal in forests also found in open bushy areas, grasslands, plantations and gardens. *Trimeresurus* snakes feed on small mammals (rats and mice), birds, lizards and frogs. Four of *Trimeresurus* species*, T. albolabris, T. insularis, T. puniceus* and *T. purpureomaculatus*, are commonly found in Java and Sumatra islands of Indonesia. *T. albolabris* habitat is widely spread and it is known to occur in Cambodia, China, India, Laos, Myanmar, Thailand, Vietnam, Malaysia to the west part islands of Indonesia such as Sumatra, Bangka, and West Java [3]. *T. insularis* have a narrow distribution, from East Java, Bali to the Lesser Sunda Islands of Indonesia [3]. *T. puniceus* is found from the Mentawai Archipelago, Natuna Archipelago, Sumatra island to Java island in Indonesia [3]. *T. purpureomaculatus* is distributed from Myanmar, Thailand, Peninsular Malaysia, Singapore to Sumatra island in Indonesia [5]. The phylogeny of the genus *Trimeresurus* shows that *T. albolabris* is closely related to *T. insularis* and *T. purpureomaculatus* (Figure 1) [6]. Meanwhile, *T. puniceus* is distantly related to the three species. 

*T. albolabris* and *T. insularis* are very common in the lowland. It is often encountered in rice fields, gardens, and human settlements. These two species have proven to be distinct through molecular studies even though no practical diagnostic for the morphological character could be established [3]. The two species are usually recognized based on their bright green color and reddish-brown dorsal surface of the tail. Their differences were simplified based on their locality. *T. albolabris*inhabits the west part of Java and Madura island; meanwhile,*T. insularis*is found on the east part of Java, Bali, and the Lesser Sunda Islands [3].*T. puniceus*or flat-nosed pit viper is distributed from Mentawai Archipelago, Natuna Archipelago, Sumatra, to Java and small islands around West Java.*T. puniceus*from Java is identified based on its red, reddish-brown or grey body with yellowish-white specks on indistinct greyish crossbands [3]. In Java, this species lives in mountainous areas and is found in coffee and tea plantations at altitudes of 1,000 to1,200 m asl. This species is known to be terrestrial and nocturnal; it burrows into loose soil and dead leaves in the daytime, while at night, it is usually found in low bushes.*T. purpureomaculatus* have diverse body colors ranging from olive, greyish, to brownish-purple and can be found in Indonesia, particularly on Sumatra island. This species mainly lives in mangrove, coastal swamp forests, and arboreal shrubs and trees [7].

In Southeast Asia, pit vipers of the *Trimeresurus* complex significantly contribute to snakebite envenomation cases [8]. Several fatalities caused by this snake genera have been documented in Indonesia. *T. insularis* is a common cause of snakebite in lowland East Java. *T. purpureomaculatus* is considered an aggressive snake, quick to strike, and its envenomation is known to be non-fatal to humans, though it can cause uncontrolled systemic bleeding and local tissue hemorrhages [9]. The bite from all these pit vipers was reported to cause intense pain, mild to severe local swelling, and spontaneous bleeding [3]. There is no species-specific antivenom available for bite victims. The only treatment method known is a horse antivenom against Thailand *T. albolabris* venom, Thai Green Pit Viper Antivenom (GPVAV). This antivenom shows cross-reactivity and neutralization capability against Indonesian *T. insularis, T. purpureomaculatus, T. hageni* and *T. puniceus* venoms [10].

Studies have shown that phylogeny [11], ontogeny [12], diet [12-14], and geography [15] could be related to phenotypic variation in venom composition. Exploring the protein composition of snake venoms could lead to a better understanding of the prey envenomation, ecology, and their possible impact on the evolutionary history of a species. Many species of *Trimeresurus* have been studied to investigate their venomics and toxicity [16-22]. Interesting proteomics studies have been conducted on venom examination from different populations of *T. insularis* in the Lesser Sunda Islands, Indonesia [17]. The venom of Indonesian *T. puniceus* has also been recently studied to investigate its proteomic and antivenom treatment potency [22]. Although numerous studies have been dedicated to the characterization of *Trimeresurus* venoms, only a small number of studies could be found concerning the venom of *Trimeresurus*species distributed from Indonesia’s different regions, particularly for Indonesian *T. albolabris* and *T. purpureomaculatus*. Therefore, in the current study, we aimed to characterize and compare the venom composition of *T. albolabris*, *T. insularis*, *T. purpureomaculatus*, and *T. puniceus* from different regions of Indonesia. 

Based on previous venomics studies, it can be hypothesized that the venom proteomes of these four species will show high similarities of proteins particularly for the major components. We hope that the result of the study could add more information on the venom protein profiles that could contribute to the comprehension of toxin variability among the four species. The snakebite incident by these species is unavoidable, considering that these four species are common in Java and Sumatra islands, where most of the Indonesian population lives. Therefore, understanding these species’ venomics is crucial for snakebite management and antivenom development in the region. 

## Methods

### Venom collection and preparation

**Figure 2.**
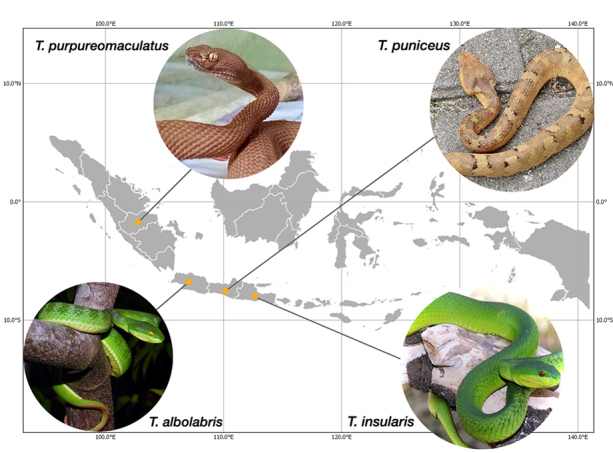
The geographical origin of the four species analyzed in the present study. All species can be easily differentiated from each other by their morphological features except for *T. albolabris* and *T. insularis*. The yellow circles represent the sample localities.

Venom samples were collected by inducing the snake to bite a sterile container, and the collected venom was immediately stored on a desiccator filled with new silica gel during transportation. Once in the laboratory, samples were stored at -20ºC after lyophilization. These procedures follow the ethical principles in animal research adopted by the World Health Organization to characterize venoms (approved by the Committee of Ethical Clearance of PT Bio Farma, 2018). After each extraction, all animals were kept alive in captivity. Venoms used in this study were pooled samples from 5 adult females and 6 adult males of *T. albolabris* from western parts of Java (SVL: 39-72 cm); 8 adult females and 5 adult males of *T. insularis* from eastern parts of Java (SVL: 40-69 cm); 7 adult females and 9 adult males of *T. puniceus* from central parts of Java (SVL: 41-70 cm); 1 adult female of *T. purpureomaculatus* from Jambi region of Sumatra island (SVL: 175 cm) (Figure 2). From all venoms collected, only about 2.4 mg dried venom of *T. albolabris*, 1.3 mg dried venom of *T. insularis*, 2.2 mg dried venom of *T. puniceus* and 1.6 mg dried venom of *T. purpureomaculatu*s were used in this study. 

### Protein concentration determination

Venom protein concentrations were determined according to the Bradford method [23], using Coomassie (Bradford) Protein Assay KIT (Thermo Scientific). Bovine serum albumin (BSA) was prepared as standard at concentrations of 2000, 1500, 1000, 750, 500, 250, 125, 25 µg/mL. Venoms were diluted six times for analysis, 250 µL of Coomassie Reagents were added to 5 µL samples and, after 10 min, absorbance was measured at 595 nm. Measurement was conducted using the Multiskan Go instrument and Thermo SkanIt RE for Multiskan Go Software version 3.2. 

### SDS-PAGE of crude venom

Initially, crude venom was fractionated using SDS-PAGE separation according to the Laemmli method [24]. Each crude venom at 60 μg and 30 μg of protein was loaded onto 15% gel and the electrophoresis was performed under reducing conditions at 80V for 2.5 h. Thermo Scientific PageRuler Prestained Protein Ladder (5 μL) was used as a marker and BSA (5 μg) as the positive control. Proteins were stained with Coomassie Brilliant Blue R-250. 

### In-gel digestion with trypsin

**Figure 3.**
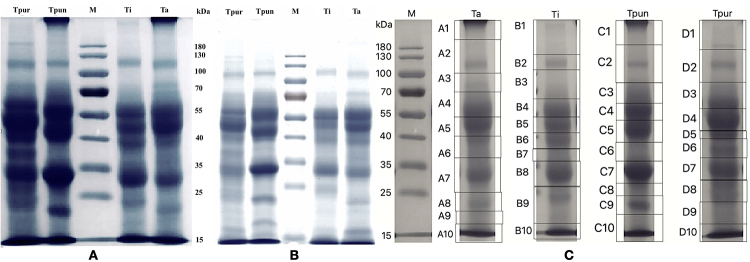
One-dimension electrophoretic profile of **(A)** 60 µg and **(B)** 30 µg of venom of T. albolabris (Ta), T. insularis (Ti), T. puniceus (Tpun), T. purpureomaculatus (Tpur). M represents the molecular weight standard in kDa. **(C)** Gel with 60 µg of crude venom was divided into sections (1-10) and further used for in-gel digestion and ESI-LCMS/MS. Ta, Ti, Tpun, Tpur showed gel sections for T. albolabris, T. insularis, T. puniceus and T. purpureomaculatus, respectively.

Gel resulting from SDS-PAGE separation loaded with 60 μg venom was divided into ten sections to have significant protein recoveries (see Figure 3). Each gel section was then excised into smaller pieces with size about 2 x 2 mm and stored in a 1.5 mL microcentrifuge tube. In-gel digestion was carried out following In-Gel Tryptic Digestion Kit Manual Instruction (Thermo Scientific). Each gel section was then destained twice, each time using 200 μL destaining solution (25 mM TEAB in 50% ACN) and incubated at 37 °C for 30 minutes. Then, the in-gel proteins were reduced with a reducing buffer (10 mM DTT) and incubated at 95 °C for 5 minutes. Then they were alkylated in the dark at room temperature for 1 hour using an alkylation buffer (500 mM IAA). After removing the alkylation buffer, gels were washed twice, each time using a destaining solution and incubated at 37 °C for 30 minutes. Gels were then dehydrated using acetonitrile at room temperature for 15 min. After slowly removing the acetonitrile solution, gels were allowed to dry for about 5-10 minutes. Afterwards, about 10 ng/μL of activated trypsin (Trypsin Gold, Mass Spectrometry Grade, Promega) were added to the gel until it was immersed and then followed by incubation at room temperature for 15 minutes. Digestion buffers were then added to the tube, and the sample was incubated at 37°C for 16-18 hours. Afterwards, to further extract peptides, 1% formic acid solution was added to the gel and vortex for 15 minutes. Samples were then clean up using Pierce C18 Spin Columns (Thermo Scientific) following the protocol and dried using a vacuum concentrator. 

### LC-MS analysis

LC-MS analysis was carried out on an Ultimate 3000 RSLC nano HPLC system connected to a QExactive Plus mass spectrometer (Thermo Fisher Scientific, USA). Samples in dissolving solution (2% ACN and 0.1% formic acid) were loaded to PepMap RSLC 50 μm × 15 cm (C18, 2 μm, 100 Å) column. Samples were eluted with a linear gradient of 80% ACN, 19.9% water, 0.1% FA (buffer B) in 99.9% water, 0.1% FA (buffer A) from 5 to 99% solution B for 30 min and 99% solution B for 10 min at 0.3 μL/min flow rate. MS data were collected in DDA mode. MS1 parameters were as follows: 70000 resolution, 310-1800 m/z scan range, maximum injection time 100 ms, AGC target 3 × 10^6^. Ions were isolated with a 1.5 m/z window targeting 10 highest intensity peaks, 4 × 10^3^ minimum AGC, preferred peptide match and isotope exclusion. Dynamic exclusion was set to 20 s. MS2 fragmentation was carried out in HCD mode at 17500 resolution with 27% NCE. Ions were accumulated for a maximum of 105 ms with a target AGC 1 × 10^5^.

### Protein identification

MS raw data files were analyzed using Proteome Discoverer Software (version 2.1; Thermo Fisher Scientific, San Jose, CA, USA) and searched against the Swiss-Prot database (2018) by selecting all proteins from the Serpentes Taxa (TaxonomyID: 8570) using MASCOT. The enzyme specificity parameter was set to “trypsin”, with two maximum missed cleavages permitted. Carbamidomethylation of cysteine was set as a static modification and oxidation of methionine. Acetyl (protein N-term) was set as a dynamic modification. The mass tolerance was 10 ppm; false discovery rate (FDR) 1%; percolator strategy; proteins must have score sequence HT > 0 and unique peptide ≥2. Proteins detected were then classified according to their protein families and their relative abundances were calculated based on their occurrence following Abidin et al. [18] using the formula:

$$\frac{No.\;of\;proteins\;(protein\;family)}{Total\;proteins\;detected\;using\;LC-MS/MS}\times 100$$

### Cluster analysis

All identified proteins were then analyzed using clustering analysis. All proteins were paired with venoms in binary form: one means presence and zero means absence. The calculation of pairwise distance matrix was performed using the Jaccard method followed with agglomerative hierarchical clustering using the hclust function in R studio to produce dendrogram [25, 26]. Heatmap visualization of all proteins was created using the ggplot2 package [27] and based on the hierarchical clustering result. We also grouped all identified proteins using the Venn diagram to visualize the number of overlap proteins between venoms and the number of exclusive proteins in each venom using the ggvenn package [28]. 

## Results

Protein content in the venom of four Trimeresurus species was determined using Bradford assay. The venom of T. albolabris contained the highest protein content of 11.1 mg/mL, followed by T. puniceus, T. insularis and T. purpureomaculatus venom with 10.7 mg/mL, 8.9 mg/mL, and 5.5 mg/mL protein, respectively. The protein profile was evaluated in SDS-PAGE gel electrophoresis using two different protein amounts of crude venom, 60 μg (Figure 3A) and 30 μg (Figure 3B). Both gel separations revealed different intensities that possibly correlated with the amount of crude venom used, where 60 μg gel showed more intense bands than 30 μg venom gel. In addition, the protein band pattern of each venom also showed different intensities that are possibly related to protein content differences. In both gels around 55-40 and 35-25 kDa protein bands, T. albolabris (Ta) and T. puniceus (Tpun) venoms showed stronger intensity compared to T. purpureomaculatus (Tpur) and T. insularis (Ti). SDS-PAGE analysis revealed that the molecular weight of major proteins in the four Trimeresurus venoms was presumably around 55-40 kDa and 35-25 kDa. Medium intensity of protein bands was also observed, approximately a band between 130-100 kDa and a band just below 25 kDa. Based on the SDS gel profile, the Tpun venom lane showed the most abundant proteins compared to other venoms, particularly for bands between 35-25 kDa and approximately below 25 kDa. For further characterization, each lane in gel with 60 μg venom was excised into ten pieces (Figure 3C). Peptides were extracted from each gel section using in-gel digestion and further subjected to LC-MS/MS analysis.

### Summary of identified protein

LC-MS/MS analysis identified 11 protein families in all venoms: snake venom metalloprotease (SVMP), C-type lectin (CTL), snake venom serine protease (SVSP), phospholipase A2 (PLA2), 5'-nucleotidase (5'-NUC), L-amino acid oxidase (LAAO), phosphodiesterase (PDE), phospholipase B (PLB), glutaminyl-peptide cyclotransferase (QPCT), amine oxidase (AO), and cysteine-rich secretory proteins (CRISP). In addition, it also identified nine protein families belonging only to several snake venoms: nerve growth factor (NGF), actin, glutathione peroxidase, aminopeptidase, endonuclease, serum albumin, transferrin, alkaline phosphatase, and cyclophilin-type PPIase. Information of all proteins identified in each snake venom is described in Additional files 1-4. In total, our venomic analysis identified 65 proteins belonging to 16 protein families in T. purpureomaculatus; 64 proteins belonging to 18 protein families in T. albolabris; 58 different proteins belonging to 14 protein families in T. puniceus; and 48 different proteins belonging to 14 protein families in T. insularis (Table 1). 

**Figure 4.**
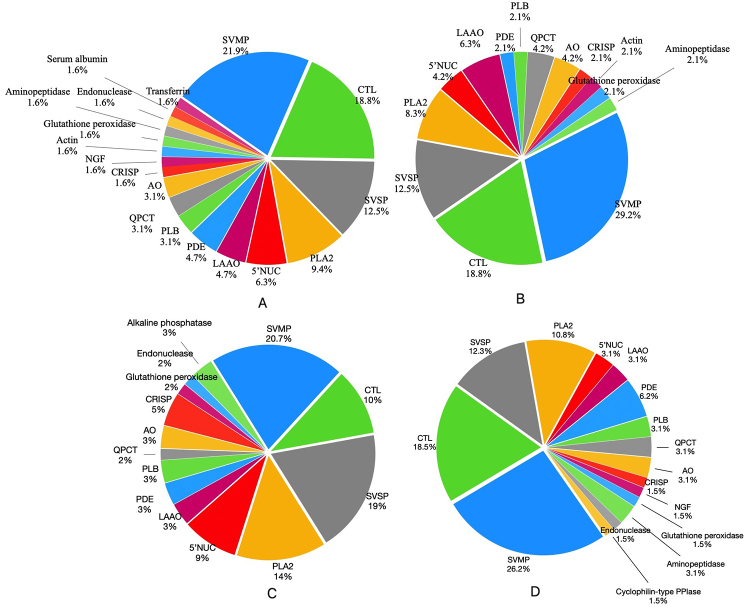
Relative occurrences of venom proteins that were detected from venoms of **(A)** T. albolabris, **(B)** T. insularis, **(C)** T. puniceus, and **(D)** T. purepureomaculatus. SVMP, snake venom metalloproteinase; SVSP, snake venom serine protease; PLA2, phospholipase A2; CTL, snake C-type lectin; CRiSP, cysteine-rich protein; LAAO, L-amino acid oxidase; PDE, phosphodiesterase; NUC, 5’-nucleotidase; endonuclease, endonuclease domain-containing 1 protein; PLB, phospholipase B; AO, amine oxidase; QPCT, glutaminyl-peptide cyclotransferase; NGF, nerve growth factor.

The major proteins composed about 60% of the total identified proteins in each venom belonged to SVMP, CTL, SVSP, and PLA2 (Figure 4). These proteins are commonly identified in Viperidae snake venoms, particularly from Trimeresurus venom (Additional files 1-4). Although with different relative abundances, the major toxin family detected in all venoms was SVMP (Table 1). There were 17 proteins identified as SVMP in Tpur, 14 proteins in Ta, 14 proteins in Ti, and 11 proteins in Tpun. Detailed analysis showed that all SVMP types belong to Viperidae venom except for one metalloproteinase in Tpur and Ta that is homolog with Hypsiglena sp. CTL was the second toxin family with most proteins identified in Ta, Ti, and Tpur venoms. However, in Tpun, the second most dominant protein family belonged to SVSP (11 proteins), almost comparable with the number of SVMP proteins identified in its venom. Another major toxin composing all Trimeresurus venom was PLA2. T. puniceus venom contained the highest variation of PLA2, in which there were eight types of proteins belonging to this protein family. Venoms of Tpur, Ta, and Ti have 7, 6, and 4 proteins of PLA2, respectively.

Other significant proteins whose presence was less than five types but identified in all venoms were 5’-NUC, LAOO, PDE, PLB, QPCT, AO, and CRISP. Tpun venom contained more various types of 5’-NUC, in which there were five types of proteins belonging to this protein family. Meanwhile, the result indicated that the relative occurrence of LAAO protein was comparable in all venom. PDE proteins were found more varied in Tpur venom with four proteins, followed by three proteins in Ta, two proteins in Tpun and one protein in Ti. PLB and QPCT protein numbers were almost comparable in all venoms, whereas the number of identified proteins as AO was similar, with two proteins in each venom. There were three proteins identified as CRISP in Tpun, while only one protein was identified as CRISP in the rest of the venom. In addition, there were several proteins identified only in specific venom. Two proteins only existed in three venom species: aminopeptidase (not detected in Tpun venom) and endonuclease (not detected in Ti). Two proteins that were identified only in two species were NGF and Actin. NGF was identified in Ta and Tpur venom, whereas actin was identified in Ta and Ti venom. Serum albumin and transferrin were detected only in Ta venom. Proteins identified only in one venom species were ALP (alkaline phosphatase) in Tpun and Cyclophilin-type PPIase in Tpur. 

**Figure 5.**
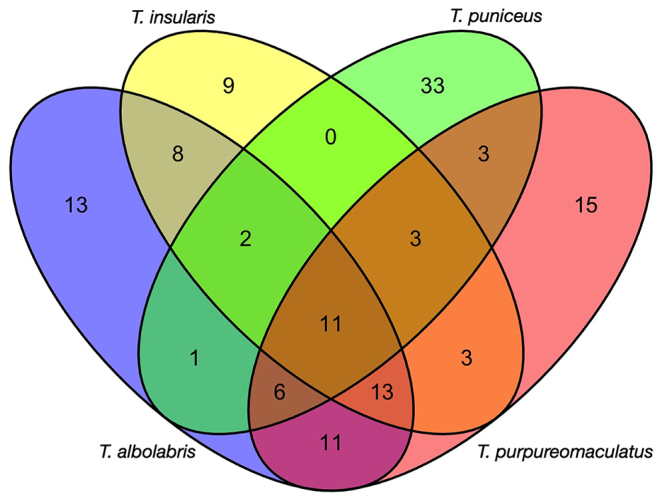
Venn diagram representing the number of proteins identified from four Trimeresurus venoms. Differently shaded areas indicate number of overlap proteins among the venoms.

**Figure 6.**
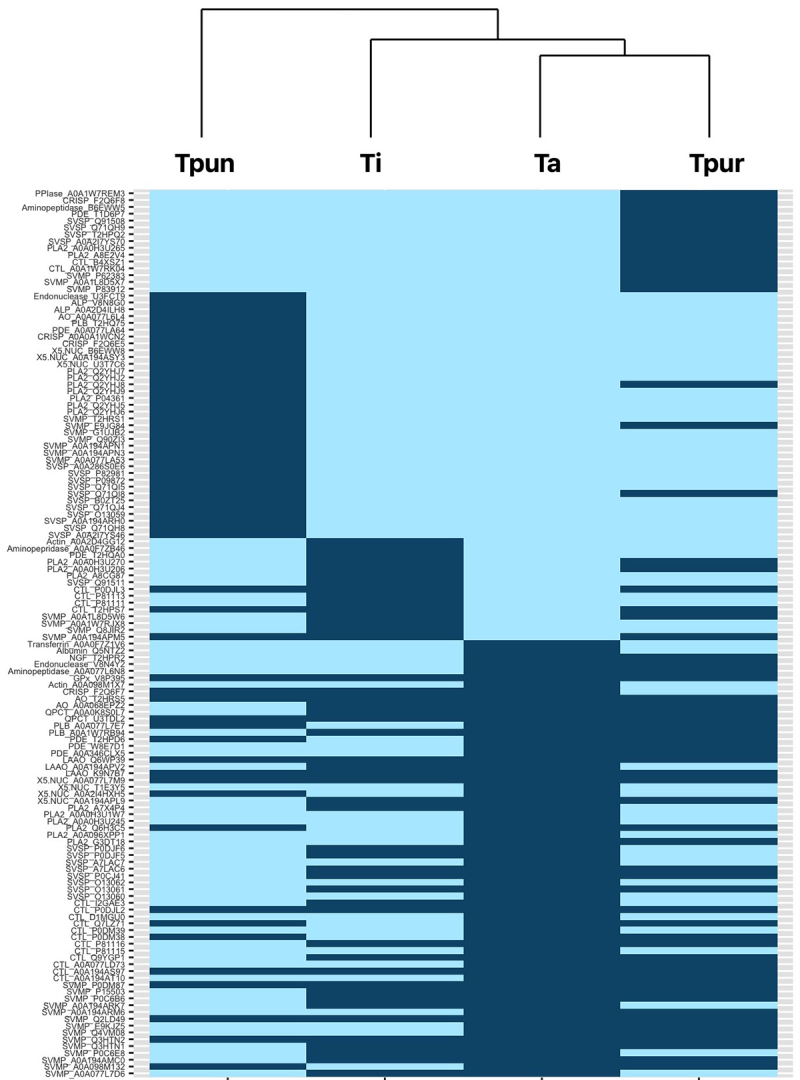
Clustering of T. albolabris (Ta), T. insularis (Ti), T. purpureomaculatus (Tpur), and T. puniceus (Tpun) venoms based on their proteomes. Proteins represented by protein family followed by the accession code. For each venom, a given protein is either present (pale blue) or absent (dark blue). SVMP, snake venom metalloproteinase; SVSP, snake venom serine protease; PLA2, phospholipase A2; CTL, snake C-type lectin; CRiSP, cysteine-rich protein; LAAO, L-amino acid oxidase; PDE, phosphodiesterase; NUC, 5’-nucleotidase; endonuclease, endonuclease domain-containing 1 protein; PLB, phospholipase B; AO, amine oxidase; QPCT, glutaminyl-peptide cyclotransferase; NGF, nerve growth factor.

Proteins commonality between venoms was analyzed using the Venn diagram (Figure 5). The comparison shows that 11 proteins were common to all venom proteomes. There were identical proteins detected in two or three venoms except for Ti and Tpun venoms which did not have common protein. Proteins uniquely expressed in Ta, Ti, Tpun, Tpur venom were 13, 9, 15, and 33 respectively. All identified proteins were grouped to all venoms using hierarchal clustering, as shown in Figure 6. Hierarchical cluster analysis of these proteins illustrated interesting patterns of similarities and differences between the venoms. It was shown that Ta and Tpur venoms belonged to one group, and they have a relatively close relationship with Ti. An apparent trend in the proteins cluster was that the venom of Tpun exhibited relatively distant relationship with the other three venoms. 

**Table 1.** Summary of protein families and the number of proteins identified from four Indonesian Trimeresurus venoms.

Protein family	*T. albolabris (Ta)*		*T. insularis (Ti)*		*T. puniceus (Tpun)*		*T. purpureomaculatus (Tpur)*	
	Protein number	Gel section	Protein number	Gel section	Protein number	Gel section	Protein number	Gel section
SVMP	14	A1-7	14	B2-9	12	C1-8	17	D1-10
CTL	12	A1-6, A8-A10	9	B4, B6-10	6	C1-2, C6	12	D2-10
SVSP	8	A1-7	6	B3-9	11	C1-10	8	D2-10
PLA2	6	A1, A3, A4, A6, A8, A10	4	B4, B8-10	8	C1-7, C9, C10	7	D2-3, D5, D8-10
5'-NUC	4	A1, A3, A4	2	B3, B6	5	C1-3, C5-7	2	D2-3, D5, D7
LAAO	3	A1, A3, A4	3	B4-5, B8, B10	2	C1, C4, C5, C7	2	D3-4
PDE	3	A1-3	1	B2, B4	2	C1-3	4	D1-3
PLB	2	A1, A4-6	1	B5-6	2	C1, C5	2	D5-7
QPCT	2	A4-5	2	B5, B6, B7, B9	1	C5-7	2	D4-7
AO	2	A1-7	2	B2-9	2	C1-8	2	D1-9
CRISP	1	A1, A7, A9, A10	1	B3	3	C1-2, C5-10	1	D8
NGF	1	A10	-	-	-	-	1	D10
Actin	1	A2	1	B3	-	-	-	-
Glutathione peroxidase	1	A8	1	B9	1	C8-9	1	D9
Aminopeptidase	1	A2	1	B5	-	-	2	D1-2
Endonuclease	1	A6	-	-	1	C7	1	D6-7
Serum albumin	1	A4	-	-	-	-	-	-
Transferrin	1	A1, A3	-	-	-	-	-	-
Alkaline phosphatase	-	-	-	-	2	A2	-	-
Cyclophilin-type PPIase	-	-	-	-	-	-	1	D9
Total	64		48		58		65	

The gel section indicated the major sites of the protein family identified in the stained SDS-PAGE gel shown in Figure 3C. SVMP, snake venom metalloproteinase; SVSP, snake venom serine protease; PLA2, phospholipase A2; CTL, snake C-type lectin; CRiSP, cysteine-rich protein; LAAO, L-amino acid oxidase; PDE, phosphodiesterase; NUC, 5’-nucleotidase; endonuclease, endonuclease domain-containing 1 protein; PLB, phospholipase B; AO, amine oxidase; QPCT, glutaminyl-peptide cyclotransferase; NGF, nerve growth factor; -, undetected.

## Discussion

The present study revealed a typical protein bands pattern of four Indonesian Trimeresurus venom and several unique bands suggesting inter-species variation in the composition of toxins. Proteomes results were generated using pooled venom of individuals from certain geographic scope, regardless of age, sex, or diet. It should be noted that these factors could influence snake venom variability as it has been shown by previous studies revealing the effect of variables such as ontogenetic [29], sex and siblings [30], or diet and prey diversity [31] on venom composition and activity. Even though it appeared that both gels have low resolutions that were possibly caused by the high amount of raw venom or the high gel concentration used in the method, the result conformed with the protein bands pattern of Trimeresurus spp. venom described in previous studies [17, 20, 21]. Therefore, it was interesting to compare and discuss the detected proteins. For instance, a previous study on the venom analysis of T. albolabris (Ta) (unknown geographical origin) using gel filtration coupled with LC-MS/MS technique reported four types of proteins [32]. A more recent study on Ta venom showed the most abundant protein families were SVMP, followed by PLA2, disintegrin, SVSP, and CTL [18]. Our study also detected similar proteins reported by both studies, although with different compositions. Major toxin components of Ta venom identified in the current study were composed of SVMP, CTL, SVSP, PLA2 except for disintegrin that was not detected. A qualitative study on Malaysian T. purpureomaculatus (Tpur) venom sample reported 13 protein families with major protein groups were SVMP, Snaclec, SVSP, LAAO [16]. Investigation of Thailand Tpur venom reported seven toxin families in which SVMP and PLA2 as the major protein groups beside other unidentified peptides and proteins [33]. More recent studies on the same species from the same geographical origin using a quantitative proteome approach showed SVMP, PLA2, CTL, and disintegrins as the major components [18]. Those studies revealed major protein families similar to our study though in different compositions. 

A study on T. insularis (Ti) venoms from eight Lesser Sunda Islands of Indonesia showed remarkable similarities of gel electrophoresis profiles and RP-HPLC patterns despite the geographical isolation [17]. The study specifically analyzed the venom toxin composition of Ti from one island and identified 18 protein/peptides families in which the major protein families were PLA2, SVSP, SVMP, and CTL. Our study on Ti venom detected 48 proteins that belong to 14 protein families. The major toxin components identified were consistent with common Trimeresurus proteomes, which are mainly composed of SVMP followed by CTL, SVSP, and PLA2. It is worth mentioning that the venom sample of Ti in this study was collected from multiple adult snakes from eastern parts of Java island. Meanwhile, the sample used in the previous research was from Sumbawa island. A recent study on Indonesian T. puniceus (Tpun) (unspecified to the exact island) showed 12 toxin families comprised of a higher abundance of PLA2 followed by SVSP, SVMP and disintegrin [22]. Our study on the Tpun venom from multiple adult snakes of central parts of Java identified 58 proteins of 14 protein families. The major toxin families detected in this study were SVMP, SVSP, PLA2, and CTL, showing an almost similar pattern with the previous study except for disintegrin. 

Figure 6 showed a hierarchical cluster visualization of all identified proteins based on their presence and absence in all venoms. The clustering result indicated that Ta, Tpur, and Ti venoms were more similar. Meanwhile, Tpun exhibited the most distinct venom composition. This pattern was also in parallel with the Venn diagram that showed Tpun venom contained the highest number of exclusive proteins, which was 33 proteins. Interestingly, cluster analysis of the proteins and venoms was also congruent with the Trimeresurus phylogenetic relationships (see Figure 1). These proteomes clustering of four venoms also reflected the immunoreactivity pattern of antivenom, GPVAV, shown in the previous study [10]. Based on the potencies of GPVAV in cross-neutralizing the venoms of Indonesian T. insularis, T. purpureomaculatus and T. puniceus, the study suggested that the antigenicity of venom proteins of these species was possibly related and conserved. Moreover, the study also specifically found the different activity of GPVAV in cross-neutralizing the Tpun venom. This activity implied ﻿that the venom toxins of this species could be more divergent from the other. The four venoms proteomes clustering and phylogenetic relationship possibly matched the previous study’s finding, where Tpun have a distant relationship with the other three species (see Figure 1). This finding suggested that snake venoms variability study guided by taxonomy relationship is valuable because it might serve information that impacts snakebite treatment and antivenom development. 

Protein identified in all venoms mainly belonged to Viperidae snake venoms, particularly Trimeresurus. Previous studies have reported different biological and toxic activities of Trimeresurus spp. venoms, explaining the evolutionary and clinical implications of the venom variations [17, 18, 20, 21]. Similar to other snake venoms, the toxin components of Trimeresurus venom have the primary function of weakening prey to facilitate their capture and prey digestion through various molecular mechanisms. Therefore, knowing the composition of snake venom can provide an overview of the molecular mechanisms that are significant in supporting the venom function. Several studies have described the mechanism of the typical protein families identified in the Trimeresurus venoms such as SVMP, PLA2, SVSP, CTL/Snaclec, LAAO, and CRISP. SVMPs were one of the most significant components of snake venoms and presented substantially in almost all Trimeresurus. This enzyme was known for inducing hemorrhage, edema, myonecrosis, blistering, dermonecrosis and a prominent inflammatory reaction [34]. The type of pharmacological activities caused by SVMP depended on its subclasses. For example, the SVMP P-III subclass has various activities, including hemorrhagic, apoptotic, vWF cleavage, activation of prothrombin, and activation of factor X. Its hemorrhagic effect was more potent than the SVMP P-1 subclass [35]. Many studies identified PLA2 as a significant enzyme with high relative abundance after SVMP in Trimeresurus spp. Snake venom PLA2 was known to exhibit various pharmacological effects such as initiation or inhibition of platelet aggregation [36]. However, a specific study on the structure and function of six PLA2 molecules derived from T. puniceus reported that this enzyme inhibited blood coagulation rather than platelet aggregation [37]. 

Snake venom serine proteases (SVSP) were also a significant protein group identified in all venom studied in this research, particularly Tpun venom. SVSP protein families comprise enzymes that catalyze a broad range of reactions involving the coagulation cascade, kallikrein-kinin, fibrinolytic, complement systems, endothelial cells, and blood platelets [38]. One subgroup of SVSPs that has been a subject of many intense studies was thrombin-like SVSPs (TL-SVSPs) that were functionally similar to thrombin in several ways [38]. The thrombin-like enzyme (TLE) detected in our study mostly resembled the TLE of T. albolabris, except for the TLE in T. puniceus that was also homologous with contortrixobin from Agkistrodon contortrix contortrix. Another SVSP form identified in this study included plasminogen activators and fibrinogenase. Our result showed that Tpun venom contained the most various and different types of SVSP. From eleven SVSP identified in Tpun, only one protein shared a similar homology with other SVSPs in the other three venoms (see Additional files 1- 4). One of the non-enzymatic proteins frequently identified in Trimeresurus spp. was C-type lectin protein (CTL). This protein is a calcium-dependent protein group that binds sugar residues and commonly targets coagulation factors [39, 40]. CTL of Ta venom could cause platelet dysfunction by binding directly to the von Willebrand factor receptor, facilitating platelet agglutination, and potentiating hemorrhagic activity [41]. CTLs were found significant in all Trimeresurus venoms studied in this research and conformed with previous studies [16-21]. Furthermore, CTLs in all venoms were mostly homologs with other Trimeresurus (see Additional files 1 to 4).

5’-NUC and LAAO were detected in all venoms, though with different relative occurrences. LAAO is an enzyme that occurs widely in snake venoms and exhibits moderate lethal toxicity. These lethal toxicity ranges from edema-inducing, platelet aggregation-inducing or inhibiting, apoptosis-inducing, also antibacterial, anticoagulant, anti-HIV effects, and other physiological effects [42]. The enzyme 5’-NUC is hydrolytic, and its pharmacological roles in venoms are not yet clearly defined. This enzyme group is involved in inhibiting platelet aggregation and blood coagulation [43]. 5’NUC protein was more abundant in Tpun and Ta venoms than the other two venoms. Minor proteins detected in all venoms of this study that accounted for about or mostly below 6 % were PDE, PLB, QPCT, AO, CRiSP, and Glutathione peroxidase. The detailed activity of CRiSP was still unknown, although it was associated with blockage of several ion channels and inhibited smooth muscle contraction, which exhibited neurotoxicity [44]. PDEs from snake venom have several roles, such as metabolizing and regulating nucleotides, leading to cardiac arrest, strokes, hypertension, and atherosclerosis [45]. In this study, venom from Ta and Tpur has more PDE types than Tpun and Ti. PLB is an enzyme detected in all venoms with low relative occurrence. The biological function of PLB in snake venoms was still unclear, though some studies have demonstrated its hemolytic activity in vitro and characterized its structure [46, 47]. Glutaminyl-peptide cyclotransferase (QPCT) is an enzyme detected in this study and also identified in Sumbawa T. insularis and Malaysia T. purpureomaculatus venom [16, 17]. This toxin indirectly contributes to venom toxicity through posttranslational modification of venom proteins. It also involves in the N-terminal glutamine cyclization that induces toxin maturation, exopeptidase degradation protection, and assists proper protein conformation [45]. It should be noted that the multiple forms of 5’-NUC, LAAO, PLB, and QPCT as detected in our study were uncommon. In most pit viper venoms, these proteins were usually present only in one or two forms. Another minor snake venom component identified in all venoms was amine oxidase (AO) which was rarely identified in other Trimeresurus venom except in Sumbawa T. insularis venom [17]. 

In addition, there were also several minor proteins identified only in specific venoms. For example, NGF was detected only in Ta and Tpur venom. Aminopeptidase was identified in all venoms except in Tpun and was also found in Thailand T. albolabris and Sumbawa T. insularis [17, 18]. Another enzyme that identified in three snake venoms was endonuclease, which was undetected only in Ti venom. The small abundance of this enzyme was identified in the venom of Malaysia Tpur, Thailand Ta, and Malaysia T. nebularis [18, 19]. The absence of endonuclease was also observed in Sumbawa T. insularis venom [17]. The role of endonucleases in the snake venom is still unclear, though it is known to be composed of DNases that hydrolyze DNA and RNases that hydrolyze RNA [48]. Proteins identified only in Ta were serum albumin and transferrin, which were involved in molecular processes such as ion binding and transportation within cells [49, 50]. Another unique enzyme identified only in one venom was alkaline phosphatase in Tpun and Peptidyl-prolyl cis-trans isomerase in Tpur. This enzyme role is still unclear, but its function is possibly related to the biological process of protein folding [49, 50]. Most of these minor toxin roles are not yet clearly understood. Even though actin, serum albumin, transferrin, and cyclophilin-type PPIase identified in this study shared similar homology with snake venom proteins, it should be noted that these proteins are uncommon snake venom components. There is a possibility that the uncommon proteins were contaminants of cellular debris from the venom gland due to the lack of raw venom preparation. Further investigation of the structure and functions of these minor toxins could deepen our comprehension of snake venom variability. 

## Conclusions

This study presents a comparative analysis of venom of four Trimeresurus species commonly found and distributed in Indonesia's major islands, namely, T. albolabris, T. insularis, T. puniceus from Java island and T. purpureomaculatus from Sumatra island. Overall, the venom composition of these four snake species appears to have a high similarity of proteins, comprising at least four major protein families that correlate with the toxin properties of the venoms. However, the venomics analysis also shows different relative occurrences of the toxin components and reveals several minor proteins. A comparison of the venom proteomes shows 11 common proteins that belong to all venoms. T. puniceus venom has the highest number of unique proteins and a distant relationship with the other three venoms. Cluster analysis of the proteins and venoms was parallel with the species phylogenetic relationships. This study adds more information on interspecies venomics variability that might contribute to the effective snakebite treatment and to the findings of the optimum heterologous antivenom.

## Supplementary material

The following online material is available for this article:

Additional file 1. Summary of detected proteins from the in-gel digestion proteins (gel sections 1-10) of T. albolabris venom by LC-MS/MS analysis.

Additional file 2. Summary of detected proteins from the in-gel digested proteins (gel sections 1-10) of T. insularis venom by LC-MS/MS analysis.

Additional file 3. Summary of detected proteins from the in-gel digested proteins (gel sections 1-10) of T. puniceus venom by LC-MS/MS analysis.

Additional file 4. Summary of detected proteins from the in-gel digested proteins (gel sections 1-10) of T. purpureomaculatus venom by LC-MS/MS analysis.
