# Supplementary material for: Venom composition of Trimeresurus albolabris, T. insularis, T. puniceus and T. purpureomaculatus from Indonesia
Source: J Venom Anim Toxins Incl Trop Dis. 2022 Jul 6;28:e20210103. doi: 10.1590/1678-9199-JVATITD-2021-0103 (PMC9261747; doi:10.1590/1678-9199-JVATITD-2021-0103)
Supplement: Additional file 1. [file 1678-9199-jvatitd-28-e20210103-s1.pdf]

J Venom Anim Toxins incl Trop Dis
https://doi.org/10.1590/1678-9199-JVATITD-2021-0103

Supplementary Material to "Venom composition of *Trimeresurus albolabris, T. insularis, T. puniceus* and *T. purpureomaculatus* from Indonesia"

Additional file 1. Summary of detected proteins from the in-gel digestion proteins (gel sections 1–10) of *T. albolabris* venom by LC-MS/MS analysis.

No	Accession	Protein family	Protein name	Organism	Gel section	Unique peptide
1	A0A077L7D6	SVMP	Metalloprotease P-IIIa (Fragment)	*Protobothrops elegans*	A5	2
2	A0A098M132	*PIII Class*	Metalloproteinase (Type III) 8c	*Hypsiglena* sp.	A1-A4	2
3	A0A194AMC0		Metalloproteinase type III 9b	*Agkistrodon piscivorus*	A1, A2, A4, A5, A6	2
4	P0C6E8		Zinc metalloproteinase/disintegrin (Fragment)	*Trimeresurus gramineus*	A6	2
5	Q3HTN1		Zinc metalloproteinase-disintegrin-like stejnihagin-A	*Trimeresurus stejnegeri*	A2, A4	2,6
6	Q3HTN2		Zinc metalloproteinase-disintegrin-like stejnihagin-B	*Trimeresurus stejnegeri*	A4	2
7	Q4VM08		Zinc metalloproteinase-disintegrin-like VLAIP-A	*Macrovipera lebetina*	A4	2
8	E9KJZ5		Group III snake venom metalloproteinase	*Echis ocellatus*	A3	2
9	Q2LD49	*PII Class*	Zinc metalloproteinase-disintegrin-like TSV-DM	*Trimeresurus stejnegeri*	A1-6	4,5,7,8
10	A0A194ARM6		Metalloproteinase type II 4	*Agkistrodon piscivorus*	A1	2
11	A0A194ARK7		Metalloproteinase type III 8	*Agkistrodon piscivorus*	A5	2
12	P0C6B6		Zinc metalloproteinase homolog-disintegrin albolatin	*Trimeresurus albolabris*	A1, A4, A5, A6	2,3,4
13	P15503		Zinc metalloproteinase/disintegrin	*Trimeresurus gramineus*	A1	2
14	P0DM87		Zinc metalloproteinase-disintegrin stejnitin	*Trimeresurus stejnegeri*	A1, A6, A7	3
15	A0A194AT10	CTL	C-type lectin 10a	*Sistrurus miliarius barbouri*	A1	5
16	A0A194AS97		C-type lectin 10b	*Sistrurus miliarius barbouri*	A10	4
17	A0A077LD73		C-type lectin F IX/X B	*Protobothrops flavoviridi*	A1, A10	2
18	Q9YGP1		C-type lectin TsL	*Trimeresurus stejnegeri*	A1, A10	3
19	P81115		Snaclec alboaggregin-B subunit alpha	*Trimeresurus albolabris*	A1, A2, A3, A4, A6, A10	2
20	P81116		Snaclec alboaggregin-B subunit beta	*Trimeresurus albolabris*	A5, A8, A9	2,3
21	P0DM38		Snaclec alboaggregin-D subunit alpha	*Trimeresurus albolabris*	A1, A2, A4, A5, A6, A9, A10	2,3,5
22	P0DM39		Snaclec alboaggregin-D subunit beta	*Trimeresurus albolabris*	A1	2
23	Q7LZ71		Snaclec coagulation factor IX-binding protein subunit A	*Protobothrops flavoviridis*	A1, A10	2
24	D1MGU0		Snaclec jerdonibitin subunit alpha	*Protobothrops jerdonii*	A1	2
25	P0DJL2		Snaclec purpureotin subunit alpha	*Trimeresurus purpureomaculatus*	A1, A2, A3, A6, A9, A10	2,3,4,5,6
26	I2GAE3		CTLPs subunit alpha (Fragment)	*Gloydius halys*	A1	2
27	O13060	SVSP	Snake venom serine protease 2A homolog	*Trimeresurus gramineus*	A6	2

No	Accession	Protein family	Protein name	Organism	Gel section	Unique peptide
28	O13061		Snake venom serine protease 2B	*Trimeresurus gramineus*	A1, A2, A4, A5, A6	2,4
29	O13062		Snake venom serine protease 2C	*Trimeresurus gramineus*	A1	2
30	P0CJ41		Alpha-fibrinogenase albofibrase	*Trimeresurus albolabris*	A1-7	4,5,7,8,9
31	A7LAC6		Thrombin-like enzyme 1	*Trimeresurus albolabris*	A3-4	2,3
32	A7LAC7		Thrombin-like enzyme 2	*Trimeresurus albolabris*	A4-5	2,4
33	P0DJF5		Venom plasminogen activator GPV-PA	*Trimeresurus albolabris*	A5-6	3,5
34	P0DJF6		Thrombin-like enzyme chitribrisin	*Trimeresurus albolabris*	A4	2
35	G3DT18	PLA2	Acidic phospholipase A2 BmooPLA2	*Bothrops moojeni*	A1	2
36	A0A096XPP1		Acidic phospholipase A2 isoform a	*Gloydius intermedius*	A3	2
37	Q6H3C5		Basic phospholipase A2 Ts-G6D49	*Trimeresurus stejnegeri*	A10	2
38	A0A0H3U245		Phospholipase A2	*Trimeresurus albolabris*	A1, A4, A8, A10	3,4,5
39	A0A0H3U1W7		Phospholipase A2	*Trimeresurus cardamomensis*	A1	4
40	A7X4P4		PLA2(IIA)-Aze2	*Azemiops feae*	A6	2
41	A0A194APL9	5'-NUC	Snake venom 5'-nucleotidase	*Agkistrodon piscivorus*	A3	4
42	A0A2I4HXH5		Venom 5'-nucleotidase	*Naja atra*	A3	2
43	T1E3Y5		Ecto-5'-nucleotidase	*Crotalus horridus*	A3	2
44	A0A077L7M9		5-nucleotidase	*Protobothrops flavoviridis*	A1, A4	2,3
45	K9N7B7	LAAO	L-amino acid oxidase Cdc18 (Fragment)	*Crotalus durissus cumanensi*	A1, A4	2
46	A0A194APV2		L-amino acid oxidase	*Agkistrodon piscivorus*	A3	2
47	Q6WP39		L-amino-acid oxidase	*Trimeresurus stejnegeri*	A1, A4	2
48	A0A346CLX5	PDE	Phosphodiesterase (Fragment)	*Borikenophis portoricensis*	A2, A3	2
49	W8E7D1		Phosphodiesterase	*Macrovipera lebetina*	A2	4
50	T2HPD6		Phosphodiesterase	*Protobothrops flavoviridis*	A1, A2, A3	3,5,6
51	A0A1W7RB94	PLB	Phospholipase B-like	*Crotalus adamanteus*	A5	3
52	A0A077L7E7		Phospholipase B-like	*Protobothrops elegans*	A1, A4, A6	3,6,9
53	U3TDL2	QPCT	Glutaminyl_cyclase (Fragment)	*Ovophis okinavensis*	A5	8
54	A0A0K8S0L7		Glutaminyl-peptide cyclotransferases	*Crotalus horridus*	A4	12
55	A0A068EPZ2	AO	Amine oxidase	*Gloydius intermedius*	A1, A2, A4, A6	3,4
56	T2HRS5		Amine oxidase	*Protobothrops flavoviridis*	A1-7	2,5,6,7,9
57	F2Q6F7	CRISP	Cysteine-rich seceretory protein Ts-CRPYa	*Trimeresurus stejnegeri*	A1, A7, A9, A10	2
58	A0A098M1X7	Actin	Actin, alpha 2, smooth muscle	*Hypsiglena* sp.	A2	4
59	V8P395	GPx	Glutathione peroxidase (Fragment)	*Ophiophagus hannah*	A8	3
60	A0A077L6N8	Aminopeptidase	Aminopeptidase	*Protobothrops elegans*	A2	7
61	V8N4Y2	Endonuclease	Endonuclease domain-containing 1 protein	*Ophiophagus hannah*	A6	2
62	T2HPR2	NGF	Nerve growth factor	*Ovophis okinavensis*	A10	3
63	Q5NTZ2	Albumin	Serum albumin	*Protobothrops flavoviridis*	A4	5
64	A0A0F7Z1V6	Transferrin	Transferrin	*Crotalus adamanteus*	A1, A3	3,5

SVMP, snake venom metalloproteinase; SVSP, snake venom serine protease; PLA2, phospholipase A2; CTL, snake C-type lectin; CRISP, cysteine-rich protein; LAAO, L-amino acid oxidase; PDE, phosphodiesterase; 5'-NUC, 5'-nucleotidase; endonuclease, endonuclease domain-containing 1 protein; PLB, phospholipase B; AO, amine oxidase; QPCT, glutaminyl-peptide cyclotransferase; NGF, nerve growth factor; GPx, gluthathione peroxidase
