# Supplementary material for: Venom composition of Trimeresurus albolabris, T. insularis, T. puniceus and T. purpureomaculatus from Indonesia
Source: J Venom Anim Toxins Incl Trop Dis. 2022 Jul 6;28:e20210103. doi: 10.1590/1678-9199-JVATITD-2021-0103 (PMC9261747; doi:10.1590/1678-9199-JVATITD-2021-0103)
Supplement: Additional file 2. [file 1678-9199-jvatitd-28-e20210103-s2.pdf]

J Venom Anim Toxins incl Trop Dis
https://doi.org/10.1590/1678-9199-JVATITD-2021-0103

Supplementary Material to "Venom composition of *Trimeresurus albolabris, T. insularis, T. puniceus* and *T. purpureomaculatus* from Indonesia"

Additional file 2. Summary of detected proteins from the in-gel digested proteins (gel sections 1–10) of *T. insularis* venom by LC-MS/MS analysis.

No	Accession	Protein family	Protein name	Organism	Gel section	Unique peptide
1	A0A077L7D6	SVMP	Metalloprotease P-IIIa (Fragment)	*Protobothrops elegans*	B5-6	2
2	A0A194AMC0	*PIII Class*	Metalloproteinase type III 9b	*Agkistrodon piscivorus*	B2, B5, B7, B8, B9	2-3
3	A0A194ARK7		Metalloproteinase type III 8	*Agkistrodon piscivorus*	B5	2
4	A0A194APM5		Metalloproteinase type III 9b	*Sistrurus tergeminus*	B4	2
5	P0C6E8		Zinc metalloproteinase/disintegrin (Fragment)	*Trimeresurus gramineus*	B8	2
6	Q8JIR2		Zinc metalloproteinase/disintegrin-like HR1a	*Protobothrops flavoviridis*	B4	2
7	Q3HTN1		Zinc metalloproteinase-disintegrin-like stejnihagin-A	*Trimeresurus stejnegeri*	B4-6	2-4
8	Q3HTN2		Zinc metalloproteinase-disintegrin-like stejnihagin-B	*Trimeresurus stejnegeri*	B3	2
9	Q2LD49		Zinc metalloproteinase-disintegrin-like TSV-DM	*Trimeresurus stejnegeri*	B2-9	2-7
10	A0A1W7RJX8	*PII Class*	Metalloproteinase (Type II) 1	*Agkistrodon contortrix*	B9	2
11	P0C6B6		Zinc metalloproteinase homolog-disintegrin albolatin	*Trimeresurus albolabris*	B6-7	2-3
12	P15503		Zinc metalloproteinase/disintegrin	*Trimeresurus gramineus*	B7	2
13	P0DM87		Zinc metalloproteinase-disintegrin stejnitin	*Trimeresurus stejnegeri*	B8	3
14	A0A1L8D5W6		Snake venom metalloproteinase	*Bothrops atrox*	B5	2
15	A0A194AS97	CTL	C-type lectin 10b	*Sistrurus miliarius barbouri*	B7-10	3-4
16	T2HPS7		C-type lectin beta subunit (Fragment)	*Protobothrops flavoviridis*	B9	2
17	Q9YGP1		C-type lectin TsL	*Trimeresurus stejnegeri*	B10	3
18	P81111		Snaclec alboaggregin-A subunit alpha	*Trimeresurus albolabris*	B10	2
19	P81113		Snaclec alboaggregin-A subunit beta	*Trimeresurus albolabris*	B7-10	2-5
20	P81116		Snaclec alboaggregin-B subunit beta	*Trimeresurus albolabris*	B4, B7	2
21	P0DJL2		Snaclec purpureotin subunit alpha	*Trimeresurus purpureomaculatus*	B6, B7, B10	2-3
22	P0DJL3		Snaclec purpureotin subunit beta	*Trimeresurus purpureomaculatus*	B8-10	2-4
23	I2GAE3		CTLPs subunit alpha (Fragment)	*Gloydius halys*	B10	2
24	O13061	SVSP	Snake venom serine protease 2B	*Trimeresurus gramineus*	B5-7	3-5
25	Q91511		Beta-fibrinogenase mucrofibrase-5	*Protobothrops mucrosquamatus*	B8	3
26	A7LAC6		Thrombin-like enzyme 1	*Trimeresurus albolabris*	B4-8	2-7
27	P0DJF6		Thrombin-like enzyme chitribrisin	*Trimeresurus albolabris*	B3	2
28	P0CJ41		Alpha-fibrinogenase albofibrase	*Trimeresurus albolabris*	B4-9	2-7
29	P0DJF5		Venom plasminogen activator GPV-PA	*Trimeresurus albolabris*	B6-8	2

No	Accession	Protein family	Protein name	Organism	Gel section	Unique peptide
30	A8CG87	PLA2	Acidic phospholipase A2 Drk-a2	*Daboia russelii*	B9	2
31	A0A0H3U206		Phospholipase A2	*Trimeresurus albolabris*	B9, B10	4-6
32	A0A0H3U270		Phospholipase A2	*Trimeresurus erythrurus*	B10	2
33	A7X4P4		PLA2(IIA)-Aze2	*Azemiops feae*	B4, B8	2
34	K9N7B7	LAAO	L-amino acid oxidase Cdc18 (Fragment)	*Crotalus durissus cumanensis*	B5	2
35	A0A194APV2		L-amino acid oxidase	*Agkistrodon piscivorus*	B10	2
36	Q6WP39		L-amino-acid oxidase	*Trimeresurus stejnegeri*	B4, B5, B8	2
37	A0A077L7M9	5'-NUC	5-nucleotidase	*Protobothrops flavoviridis*	B3, B6	2-4
38	A0A194APL9		Snake venom 5'-nucleotidase	*Agkistrodon piscivorus*	B3	3
39	A0A068EPZ2	AO	Amine oxidase	*Gloydius intermedius*	B3, B4, B5, B7, B8	3-4
40	T2HRS5		Amine oxidase	*Protobothrops flavoviridis*	B2-9	2-8
41	U3TDL2	QPCT	Glutaminyl_cyclase (Fragment)	*Ovophis okinavensi*	B6, B7, B9	2-6
42	A0A0K8S0L7		Glutaminyl-peptide cyclotransferases	*Crotalus horridus*	B5	10
43	T2HQA0	PDE	Phosphodiesterase	*Protobothrops flavoviridis*	B2, B4	2-4
44	A0A1W7RB94	PLB	Phospholipase B-like	*Crotalus adamanteus*	B5-B6	10-11
45	F2Q6F7	CRISP	Cysteine-rich seceretory protein Ts-CRPYa	*Trimeresurus stejnegeri*	B3	2
46	A0A0F7ZB46	Aminopeptidase	Aminopeptidase	*Crotalus adamanteus*	B5	5
47	V8P395	GPx	Glutathione peroxidase (Fragment)	*Ophiophagus hannah*	B9	3
48	A0A2D4GG12	Actin	Uncharacterized protein	*Micrurus corallinus*	B3	4

SVMP, snake venom metalloproteinase; SVSP, snake venom serine protease; PLA2, phospholipase A2; CTL, snake Cc-type lectin; CRiSP, cysteine-rich protein; LAAO, L-amino acid oxidase; PDE, phosphodiesterase; NUC, 5'-nucleotidase; endonuclease, endonuclease domain-containing 1 protein; PLB, phospholipase B; AO, amine oxidase; QPCT, glutaminyl-peptide cyclotransferase; NGF, nerve growth factor; GPx, gluthathione peroxidase
