# Supplementary material for: Venom composition of Trimeresurus albolabris, T. insularis, T. puniceus and T. purpureomaculatus from Indonesia
Source: J Venom Anim Toxins Incl Trop Dis. 2022 Jul 6;28:e20210103. doi: 10.1590/1678-9199-JVATITD-2021-0103 (PMC9261747; doi:10.1590/1678-9199-JVATITD-2021-0103)
Supplement: Additional file 3. [file 1678-9199-jvatitd-28-e20210103-s3.pdf]

J Venom Anim Toxins incl Trop Dis
https://doi.org/10.1590/1678-9199-JVATITD-2021-0103

Supplementary Material to "Venom composition of *Trimeresurus albolabris, T. insularis, T. puniceus* and *T. purpureomaculatus* from Indonesia"

Additional file 3. Summary of detected proteins from the in-gel digested proteins (gel sections 1–10) of *T. puniceus* venom by LC-MS/MS analysis.

No	Accession	Family name	Protein name	Organism	Gel section	Unique peptide
1	A0A2I7YS46	SVSP	Serine endopeptidase	*Crotalus lepidus*	C6	2
2	Q71QH8		Serine protease CL4	*Trimeresurus stejnegeri*	C5	2
3	A0A194ARH0		Serine proteinase 7	*Agkistrodon piscivorus*	C1- C6	2
4	O13059		Snake venom serine protease 1	*Trimeresurus gramineus*	C2-C4, C6-C10	2-4
5	Q71QJ4		Snake venom serine protease homolog KN4	*Trimeresurus stejnegeri*	C4-C5	2
6	B0ZT25		Snake venom serine protease homolog	*Protobothrops jerdonii*	C1, C6	2
7	Q71QI8		Snake venom serine protease KN10	*Trimeresurus stejnegeri*	C6	2
8	Q71QI5		Snake venom serine protease KN3	*Trimeresurus stejnegeri*	C7	2
9	P09872		Protein C activator	*Agkistrodon contortrix contortrix*	C6	2
10	P82981		Thrombin-like enzyme contortrixobin	*Agkistrodon contortrix contortrix*	C7-C8	2-3
11	A0A286S0E6		Venom plasminogen activator 2	*Gloydius intermedius*	C5-C6	2
12	A0A077LA53	SVMP	Metalloprotease P-IIIc (Fragment)	*Protobothrops elegans*	C1, C2, C4	2
13	A0A098M132	*PIII Class*	Metalloproteinase (Type III) 8c	*Hypsiglena* sp.	C2-C4	2
14	A0A194APN3		Metalloproteinase type III 4c	*Sistrurus tergeminus*	C2-C3	2
15	A0A194APN1		Metalloproteinase type III 7	*Agkistrodon piscivorus*	C2-C4, C8	2
16	A0A194APM5		Metalloproteinase type III 9b	*Sistrurus tergeminus*	C7	2
17	Q90ZI3		Zinc metalloproteinase-disintegrin-like HV1	*Protobothrops flavoviridis*	C1, C2, C4, C5	2-5
18	Q3HTN2		Zinc metalloproteinase-disintegrin-like stejnihagin-B	*Trimeresurus stejnegeri*	C4	2
19	G1UJB2		Flavorase	*Protobothrops flavoviridis*	C4-C6	2
20	E9JG84	*PII Class*	Metalloproteinase	*Echis coloratus*	C2-C4	2
21	T2HRS1		p-II metalloprotease (Fragment)	*Protobothrops flavoviridis*	C3-C4	2
22	P0DM87		Zinc metalloproteinase-disintegrin stejnitin	*Trimeresurus stejnegeri*	C6-C7	2-3
23	Q2LD49		Zinc metalloproteinase-disintegrin-like TSV-DM	*Trimeresurus stejnegeri*	C6-C7	3
24	Q2YHJ6	PLA2	Acidic phospholipase A2 Tpu-E6a	*Trimeresurus puniceus*	C10	2
25	Q2YHJ5		Acidic phospholipase A2 Tpu-E6b	*Trimeresurus puniceus*	C1, C10	2-5
26	P04361		Basic phospholipase A2 homolog	*Agkistrodon piscivorus piscivorus*	C10	2

No	Accession	Family name	Protein name	Organism	Gel section	Unique peptide
27	Q2YHJ9		Basic phospholipase A2 homolog Tpu-K49a	*Trimeresurus puniceus*	C1, C7, C10	2-4
28	Q2YHJ8		Basic phospholipase A2 homolog Tpu-K49b	*Trimeresurus puniceus*	C1- C6, C9, C10	2-5
29	Q2YHJ2		Basic phospholipase A2 Tbo-G6D49	*Trimeresurus borneensis*	C2-C4, C6, C10	2
30	Q2YHJ7		Basic phospholipase A2 Tpu-G6D49	*Trimeresurus puniceus*	C1	2
31	Q6H3C5		Basic phospholipase A2 Ts-G6D49	*Trimeresurus stejneger*	C1, C10	2
32	A0A194AS97	CTL	C-type lectin 10b	*Sistrurus miliarius barbouri*	C1, C2, C6, C10	2-3
33	T2HPS7		C-type lectin beta subunit (Fragment)	*Protobothrops flavoviridis*	C1, C10	2
34	P0DM38		Snaclec alboaggregin-D subunit alpha	*Trimeresurus albolabris*	C10	2
35	Q7LZ71		Snaclec coagulation factor IX-binding protein subunit A	*Protobothrops flavoviridis*	C1, C10	2-4
36	P0DJL2		Snaclec purpureotin subunit alpha	*Trimeresurus purpureomaculatus*	C10	6
37	P0DJL3		Snaclec purpureotin subunit beta	*Trimeresurus purpureomaculatus*	C10	5
38	U3T7C6	5'-NUC	5'-nucleotidase (Fragment)	*Ovophis okinavensis*	C6	5
39	A0A077L7M9		5-nucleotidase	*Protobothrops flavoviridis*	C5	4
40	A0A194ASY3		Ecto-5'-nucleotidase	*Sistrurus miliarius barbouri*	C1-C3	3-6
41	B6EWW8		Snake venom 5'-nucleotidase	*Gloydius brevicaudus*	C1-C3, C7	2
42	A0A2I4HXH5		Venom 5'-nucleotidase	*Naja atra*	C2-C3	2
43	F2Q6E5	CRISP	Cysteine-rich seceretory protein Ch-CRPKa (Fragment)	*Crotalus horridus*	C2, C6, C7, C9, C10	2
44	F2Q6F7		Cysteine-rich seceretory protein Ts-CRPYa	*Trimeresurus stejnegeri*	C5	2
45	A0A0A1WCN2		Cysteine-Rich Secretory Protein B	*Echis coloratus*	C1, C8, C9	2
46	K9N7B7	LAAO	L-amino acid oxidase Cdc18 (Fragment)	*Crotalus durissus cumanensis*	C4	2
47	Q6WP39		L-amino-acid oxidase	*Trimeresurus stejnegeri*	C1, C4, C5, C7	2
48	A0A077LA64	PDE	Phosphodiesterase	*Protobothrops elegans*	C1-C3	2-3
49	T2HPD6		Phosphodiesterase	*Protobothrops flavoviridis*	C2	3
50	T2HQ75	PLB	Phospholipase B-like	*Ovophis okinavensis*	C5	2
51	A0A077L7E7		Phospholipase B-like	*Protobothrops elegans*	C1, C5	2-3
52	A0A077L6L4	AO	Amine oxidase	*Protobothrops elegans*	C1, C4	2
53	T2HRS5		Amine oxidase	*Protobothrops flavoviridis*	C1-C8	2-4
54	A0A2D4ILH8	ALP	Alkaline phosphatase	*Micrurus lemniscatus lemniscatus*	C2	2
55	V8N8G0		Alkaline phosphatase (Fragment)	*Ophiophagus hannah*	C2	3
56	U3FCT9	Endonuclease	Endonuclease domain-containing 1 protein	*Micrurus fulvius*	C7	2
57	U3TDL2	QPCT	Glutaminyl_cyclase (Fragment)	*Ovophis okinavensis*	C5-C7	3-7
58	V8P395	GPX	Glutathione peroxidase (Fragment)	*Ophiophagus hannah*	C8-C9	3

SVMP, snake venom metalloproteinase; SVSP, snake venom serine protease; PLA2, phospholipase A2; CTL, snake C-type lectin; CRISP, cysteine-rich protein; LAAO, L-amino acid oxidase; PDE, phosphodiesterase; NUC, 5’-nucleotidase; endonuclease, endonuclease domain-containing 1 protein; PLB, phospholipase B; AO, amine oxidase; QPCT, glutaminyl-peptide cyclotransferase; NGF, nerve growth factor; GPx, gluthathione peroxidase; ALP, alkaline phosphatase.
