# Supplementary material for: Venom composition of Trimeresurus albolabris, T. insularis, T. puniceus and T. purpureomaculatus from Indonesia
Source: J Venom Anim Toxins Incl Trop Dis. 2022 Jul 6;28:e20210103. doi: 10.1590/1678-9199-JVATITD-2021-0103 (PMC9261747; doi:10.1590/1678-9199-JVATITD-2021-0103)
Supplement: Additional file 4. [file 1678-9199-jvatitd-28-e20210103-s4.pdf]

J Venom Anim Toxins incl Trop Dis
https://doi.org/10.1590/1678-9199-JVATITD-2021-0103

Supplementary Material to "Venom composition of *Trimeresurus albolabris, T. insularis, T. puniceus* and *T. purpureomaculatus* from Indonesia"

Additional file 4. Summary of detected proteins from the in-gel digested proteins (gel sections 1–10) of *T. purpureomaculatus* venom by LC-MS/MS analysis.

No	Accession	Protein family	Protein name	Organism	Gel section	Unique peptide
1	E9KJZ5	SVMP	Group III snake venom metalloproteinase	*Echis ocellatus*	D8	2
2	A0A098M132	*PIII Class*	Metalloproteinase (Type III) 8c	*Hypsiglena* sp.	D2-D6, D8	2
3	A0A194AMC0		Metalloproteinase type III 9b	*Agkistrodon piscivorus*	D5	2
4	A0A194APM5		Metalloproteinase type III 9b	*Sistrurus tergeminus*	D3, D5	2
5	Q3HTN1		Zinc metalloproteinase-disintegrin-like stejnihagin-A	*Trimeresurus stejnegeri*	D2-D9	2-7
6	Q3HTN2		Zinc metalloproteinase-disintegrin-like stejnihagin-B	*Trimeresurus stejnegeri*	D2-D6	2
7	Q4VM08		Zinc metalloproteinase-disintegrin-like VLAIP-A	*Macrovipera lebetina*	D5	2
8	E9JG84	*PII Class*	Metalloproteinase	*Echis coloratus*	D3-D4	2
9	A0A194ARM6		Metalloproteinase type II 4	*Agkistrodon piscivorus*	D7	2
10	P0C6B6		Zinc metalloproteinase homolog-disintegrin albolatin	*Trimeresurus albolabris*	D3, D5-D7, D9	2-4
11	P15503		Zinc metalloproteinase/disintegrin	*Trimeresurus gramineus*	D7, D8	2-3
12	P83912		Zinc metalloproteinase-disintegrin jerdonitin	*Protobothrops jerdonii*	D10	2
13	P0DM87		Zinc metalloproteinase-disintegrin stejnitin	*Trimeresurus stejnegeri*	D2, D5, D7, D9	2-4
14	Q2LD49		Zinc metalloproteinase-disintegrin-like TSV-DM	*Trimeresurus stejnegeri*	D1- D9	3-6
15	A0A1L8D5X7		Snake venom metalloproteinase	*Bothrops atrox*	D5	3
16	P62383		Disintegrin trigramin-gamma	*Trimeresurus gramineus*	D10	3
17	A0A1L8D5W6		Snake venom metalloproteinase	*Bothrops atrox*	D2-D5	2-3
18	A0A194AT10	CTL	C-type lectin 10a	*Sistrurus miliarius barbouri*	D8	6
19	A0A194AS97		C-type lectin 10b	*Sistrurus miliarius barbouri*	D3, D6, D9, D10	2-4
20	A0A1W7RK04		C-type lectin 8	*Agkistrodon contortrix contortrix*	D10	2
21	T2HPS7		C-type lectin beta subunit (Fragment)	*Protobothrops flavoviridis*	D10	2
22	A0A077LD73		C-type lectin F IX/X B	*Protobothrops flavoviridis*	D10	2
23	Q9YGP1		C-type lectin TsL	*Trimeresurus stejnegeri*	D5, D7, D8, D10	2-4
24	B4XSZ1		Snaclec A16	*Macrovipera lebetina*	D10	2

No	Accession	Protein family	Protein name	Organism	Gel section	Unique peptide
25	P81116		Snaclec alboaggregin-B subunit beta	*Trimeresurus albolabris*	D6, D7	2-5
26	P0DM38		Snaclec alboaggregin-D subunit alpha	*Trimeresurus albolabris*	D2-D10	3-6
27	Q7LZ71		Snaclec coagulation factor IX-binding protein subunit A	*Protobothrops flavoviridis*	D10	3
28	P0DJL2		Snaclec purpureotin subunit alpha	*Trimeresurus purpureomaculatus*	D2, D3, D5-D10	3-10
29	P0DJL3		Snaclec purpureotin subunit beta	*Trimeresurus purpureomaculatu*	D3, D8-D10	4-9
30	G3DT18	PLA2	Acidic phospholipase A2 BmooPLA2	*Bothrops moojeni*	D5	3
31	A8E2V4		Acidic phospholipase A2	*Bothriechis schlegelii*	D10	2
32	Q2YHJ8		Basic phospholipase A2 homolog Tpu-K49b	*Trimeresurus puniceus*	D2, D10	2
33	Q6H3C5		Basic phospholipase A2 Ts-G6D49	*Trimeresurus stejnegeri*	D10	2
34	A0A0H3U206		Phospholipase A2	*Trimeresurus albolabris*	D2, D3, D8-D10	2-6
35	A0A0H3U270		Phospholipase A2	*Trimeresurus erythrurus*	D10	2
36	A0A0H3U265		Phospholipase A2	*Trimeresurus venustus*	D10	2
37	A0A2I7YS70	SVSP	Serine endopeptidase	*Crotalus scutulatus*	D2	2
38	T2HPQ2		Serine protease (Fragment)	*Ovophis okinavensis*	D7	2
39	O13061		Snake venom serine protease 2B	*Trimeresurus gramineus*	D5	3
40	Q71QI8		Snake venom serine protease KN10	*Trimeresurus stejnegeri*	D7	2
41	P0CJ41		Alpha-fibrinogenase albofibrase	*Trimeresurus albolabris*	D2-D10	4-10
42	Q71QH9		Snake venom serine protease KN14	*Trimeresurus stejnegeri*	D5	2
43	Q91508		Beta-fibrinogenase mucrofibrase-2	*Protobothrops mucrosquamatus*	D7	2
44	A7LAC6		Thrombin-like enzyme 1	*Trimeresurus albolabris*	D2-D7	5-8
45	A0A346CLX5	PDE	Phosphodiesterase (Fragment)	*Borikenophis portoricensis*	D2-D3	2
46	T1D6P7		Phosphodiesterase	*Crotalus horridus*	D1	6
47	W8E7D1		Phosphodiesterase	*Macrovipera lebetina*	D2-D3	2-6
48	T2HPD6		Phosphodiesterase	*Protobothrops flavoviridis*	D3	8
49	K9N7B7	LAAO	L-amino acid oxidase Cdc18 (Fragment)	*Crotalus durissus cumanensis*	D4	2
50	Q6WP39		L-amino-acid oxidase	*Trimeresurus stejnegeri*	D3-D4	2
51	A0A077L7M9	5'-NUC	5-nucleotidase	*Protobothrops flavoviridis*	D2-D3, D5, D7	3-6
52	A0A194APL9		Snake venom 5'-nucleotidase	*Agkistrodon piscivorus*	D3	2
53	A0A1W7RB94	PLB	Phospholipase B-like	*Crotalus adamanteus*	D5	4
54	A0A077L7E7		Phospholipase B-like	*Protobothrops elegans*	D6-D7	3-4
55	A0A068EPZ2	AO	Amine oxidase	*Gloydius intermedius*	D2-D5, D7	2-3
56	T2HRS5		Amine oxidase	*Protobothrops flavoviridis*	D1-D9	2-8
57	B6EWW5	Aminopeptidase	Aminopeptidase	*Gloydius brevicaudus*	D2	7
58	A0A077L6N8		Aminopeptidase	*Protobothrops elegans*	D1-D2	4-8
59	U3TDL2	QPCT	Glutaminyl_cyclase (Fragment)	*Ovophis okinavensis*	D4, D6-D7	3-8

No	Accession	Protein family	Protein name	Organism	Gel section	Unique peptide
60	A0A0K8S0L7		Glutaminyl-peptide cyclotransferases	*Crotalus horridus*	D5	12
61	F2Q6F8	CRISP	Cysteine-rich seceretory protein Ts-CRPYb	*Trimeresurus stejnegeri*	D8	2
62	V8P395	GPx	Glutathione peroxidase (Fragment)	*Ophiophagus hannah*	D9	2
63	A0A1W7REM3	Cyclophilin-type PPIase	Peptidyl-prolyl cis-trans isomerase	*Agkistrodon contortrix contortrix*	D9	3
64	V8N4Y2	Endonuclease	Endonuclease domain-containing 1 protein	*Ophiophagus hannah*	D6-D7	2-3
65	T2HPR2	NGF	Nerve growth factor	*Ovophis okinavensis*	D10	2

SVMP, snake venom metalloproteinase; SVSP, snake venom serine protease; PLA2, phospholipase A2; CTL, snake C-type lectin; CRiSP, cysteine-rich protein; LAAO, L-amino acid oxidase; PDE, phosphodiesterase; NUC, 5'-nucleotidase; endonuclease, endonuclease domain-containing 1 protein; PLB, phospholipase B; AO, amine oxidase; QPCT, glutaminyl-peptide cyclotransferase; NGF, nerve growth factor; GPx, gluthathione peroxidase.
